# Drug-Resistant *Candida glabrata* Infection in Cancer Patients

**DOI:** 10.3201/eid2011.140685

**Published:** 2014-11

**Authors:** Dimitrios Farmakiotis, Jeffrey J. Tarrand, Dimitrios P. Kontoyiannis

**Affiliations:** The University of Texas MD Anderson Cancer Center, Houston, Texas, USA (D. Farmakiotis, J.J. Tarrand, D.P. Kontoyiannis);; Baylor College of Medicine, Houston (D. Farmakiotis)

**Keywords:** *Candida glabrata*, candidemia, antifungal resistance, cancer, fungi

## Abstract

Cancer patients are at risk for candidemia, and increasing *Candida* spp. resistance poses an emerging threat. We determined rates of antifungal drug resistance, identified factors associated with resistance, and investigated the correlation between resistance and all-cause mortality rates among cancer patients with ≥1 *C. glabrata*–positive blood culture at MD Anderson Cancer Center, Houston, Texas, USA, during March 2005–September 2013. Of 146 isolates, 30 (20.5%) were resistant to fluconazole, 15 (10.3%) to caspofungin, and 10 (6.8%) to multiple drugs (9 caspofungin-resistant isolates were also resistant to fluconazole, 1 to amphotericin B). Independently associated with fluconazole resistance were azole preexposure, hematologic malignancy, and mechanical ventilation. Independently associated with caspofungin resistance were echinocandin preexposure, monocytopenia, and total parenteral nutrition. Fluconazole resistance was highly associated with caspofungin resistance, independent of prior azole or echinocandin use. Caspofungin resistance was associated with increased 28-day all-cause mortality rates. These findings highlight the need for good stewardship of antifungal drugs.

## Abstract 2

High rates of resistance to azoles and echinocandins emphasize the need for good antifungal stewardship.

Drug-Resistant *C. glabrata* Infection in Cancer Patients

Patients with cancer are often at risk for candidemia because of indwelling catheters, abdominal surgery, use of cytotoxic chemotherapy, parenteral nutrition, antibacterial drugs, and corticosteroids (*1*–*5*). Increasing drug resistance among *Candida* spp. poses an emerging threat to these patients. Moreover, the widespread prophylactic use of azoles in patients with hematologic malignancies and a reduced threshold for empiric initiation of antifungal treatment among critically ill patients have led to a notable shift from infections with *C.* *albicans* to infections with non-*albicans Candida* species (*2*–*4*). Among cancer patients, one of the most common *Candida* species isolated is *C. glabrata* (*3*–*5*), which is the main species exhibiting multiazole, echinocandin, and multidrug resistance (resistance to at least 2 classes of antifungal drugs) (*6*–*9*).

Recently, on the basis of the integration of epidemiologic, molecular, and limited clinical data, the Clinical Laboratory Standards Institute (CLSI) updated antifungal susceptibility break points for *Candida* spp. (*10*,*11*). According to the new definitions, rates of caspofungin nonsusceptibility among *C. glabrata* clinical isolates range from <10% (*12*) to as high as 62% (*13*). Previous use of azoles or echinocandins are strong predictors of resistance to the respective classes (*3*,*5*,*6*,*14*,*15*), but little is known about the current rates of cross-resistance between azoles and echinocandins in patients with cancer or about additional clinical factors that could be associated with resistance. In a contemporary cohort of cancer patients with *C. glabrata* fungemia, we determined rates of in vitro resistance and cross-resistance to azoles and echinocandins, identified factors associated with resistance, and investigated the association between antifungal resistance and all-cause mortality rates.

## Patients and Methods

We included patients seen at MD Anderson Cancer Center from March 2005 through September 2013, for whom ≥1 blood culture was positive for *C. glabrata* and who had symptoms, signs, or laboratory findings consistent with infection. We retrospectively reviewed electronic medical records for demographic, clinical, and laboratory data for the day of candidemia (defined as day of blood collection for culture), and we reviewed pharmacy records and clinical notes for previous use of antifungal drugs and cumulative doses. The study was approved by the MD Anderson Cancer Center institutional review board.

Isolation and identification of *C. glabrata* isolates in blood culture were performed by using standard microbiological procedures (*2*–*4*). We determined MICs of fluconazole, voriconazole, posaconazole, caspofungin, and amphotericin B by using the broth microdilution method described in CLSI M27-A documents (*10*,*16*), according to prominent (50%) reduction in turbidity and 100% growth inhibition for amphotericin B. In a subset of caspofungin-resistant isolates, we also tested in vitro susceptibility to micafungin and anidulafungin.

Susceptibility to antifungal drugs was defined according to clinical break points for *C. glabrata* (*10*,*11*); isolates for which fluconazole MIC was ≤32 mg/L were considered dose dependent, whereas those for which MIC was ≥64 mg/L were considered resistant. Because clinical break points for voriconazole and posaconazole are undefined, *C. glabrata* isolates in which MIC was >1 dilution above the epidemiologic cutoff values (0.5 and 2.0 mg/L, respectively) were considered potentially resistant. Caspofungin/anidulafungin and micafungin resistance was defined as MICs ≥0.5 mg/L and ≥0.25 mg/L, respectively (*10*,*11*). Strains for which caspofungin or anidulafungin MIC was 0.25 mg/L or micafungin MIC was 0.125 mg/L were classified as intermediate. Resistance to amphotericin B was defined as MIC ≥2 mg/L (*10*,*11*).

Continuous variables were compared by using the Student *t*-test or the Mann-Whitney U criterion for variables that were not normally distributed. Categorical variables were compared by using the χ^2^ test, Fisher exact test, and linear-by-linear associations for trend. Binary and ordinal (after testing the parallel lines assumption) logistic regression analyses were used to identify variables independently associated with fluconazole, caspofungin, and multidrug resistance. Survival curves were compared by using the log-rank test and Cox regression analysis. The proportional hazards assumption was tested graphically and by building time-dependent variables. For univariate analyses, clinically relevant parameters (p<0.1) were included at model entry. Variables were retained in the final model if p<0.05; p values >0.05 but <0.1 were noted as indicating trends. All analyses were performed by using SPSS, version 21 (IBM Corporation, Chicago, IL, USA).

## Results

### Patient Population

We studied 146 candidemia episodes (first positive blood culture per hospitalization) in 144 patients (Table 1). A second episode occurred for 2 patients, >2 months after the first episode. Most (68%) patients had solid tumors, whereas during 1999–2003, of 150 *C. glabrata* bloodstream isolates (*3*), 64 (42.6%) were from patients with solid tumors (p<0.001).

**Table 1:** Basic demographic, clinical, and laboratory characteristics for 144 cancer patients with 146 episodes of *Candida glabrata* fungemia, MD Anderson Cancer Center, Houston, Texas, USA, March 2005–September 2013*

Characteristic	No. (%)
Host	
Age, y, mean (± SD), range	55.5 (± 14.52), 12–85
Male sex	74 (51.38)
Solid tumor†	98 (68.05)
Hematologic malignancy	46 (31.95)
Leukemia	22 (15.3)
Acute myeloid leukemia	17 (11.81)
Acute lymphoblastic leukemia	5 (3.47)
Lymphoma	14 (9.72)
Multiple myeloma	4 (2.77)
Myelodysplastic syndrome	2 (1.38)
Myelohyperplastic syndrome	4 (2.77)
Hematopoietic stem cell transplantation	16 (11.11)
Clinical disease	
Intensive care unit stay	59 (40.41)
Mechanical ventilation	27 (18.49)
Presence of a central line	131 (89.72)
Total parenteral nutrition	36 (24.65)
Recent (within 1 mo before the day of candidemia) drug exposures	
Chemotherapy	69 (47.26)
Any corticosteroids	85 (58.21)
Antibacterial drugs	144 (98.63)
Azoles	44 (30.13)
Echinocandins	32 (21.91)
Laboratory findings	
Neutropenia, cells/μL	
<500	28 (19.17)
100–500	9 (6.16)
<100	19 (13.14)
Lymphopenia, cells/μL	
<500	86 (58.9)
<100	30 (20.54)
Monocytopenia, <100 cells/μL	39 (26.71)

*All parameters were present on the day of candidemia, defined as the day of blood culture collection. Data are presented as absolute numbers (%) unless otherwise indicated for normally distributed variables or median numbers (25th–75th percentile) for variables that were not normally distributed. †Tumor types were as follows: 47 (32.63%) gastrointestinal, 12 (8.33%) gynecologic, 9 (6.25%) genitourinary, 6 (4.16%) breast, 6 (4.16%) lung, 3 (2.08%) thyroid, 4 (2.77%) sarcomas, 3 (2.08%) head and neck, 2 (1.38%) central nervous system, and 6 (4.16%) other.

### Azole Resistance

Of the 146 isolates, 30 (20.5%) were resistant to fluconazole. For those 30, the voriconazole MIC was ≥1 mg/L (epidemiologic break point [EB] 0.5 mg/L) for 28 (93.3%) isolates, and the posaconazole MIC was ≥4 for 26 (86.6%) isolates and ≥2 mg/L (EB) for 29 (96.6%) isolates. For 1 isolate that was resistant to fluconazole (MIC 128 mg/L), MICs for voriconazole and posaconazole were both below the EB (0.25 and 0.5 mg/L, respectively). Therefore, 29 (96.6%) of the 30 fluconazole-resistant isolates could be characterized as multiazole resistant. A total of 20 (66.7%) fluconazole-resistant strains were isolated from patients with hematologic malignancies, and 10 (33.3%) were isolated from patients with solid tumors.

Factors significantly associated with fluconazole resistance are summarized in Table 2. The observed association of azole exposure with fluconazole resistance resulted mostly from recent administration of voriconazole; 14 (46.6%) of 30 patients from whom fluconazole-resistant isolates were obtained had received voriconazole within 1 month before the day of candidemia, as opposed to 10 (8.6%) of 116 from whom dose-dependent isolates were obtained (p<0.001). In comparison, 6 (20%) of the 30 patients from whom fluconazole-resistant isolates were obtained had received fluconazole within 1 month, as opposed to 19 (16%) of the 116 from whom fluconazole dose-dependent isolates were obtained (p = 0.639). Of the 30 patients from whom fluconazole-resistant isolates were obtained, 2 (6.6%) had received posaconazole within 1 month, as opposed to 1 (0.9%) of 116 from whom fluconazole dose–dependent isolates were obtained (p = 0.107). Factors independently associated with fluconazole resistance were recent azole exposure, hematologic malignancy, and mechanical ventilation (Table 2).

**Table 2:** Factors present at the time of candidemia and associated with fluconazole resistance, in cancer patients with *Candida glabrata* fungemia, MD Anderson Cancer Center, Houston, Texas, USA, March 2005–September 2013*

Factor	No. (%) cases				Multivariate analysis	
	Dose-dependent, n = 116	Resistant, n = 30	p value		Odds ratio (95% CI)	p value
Hematologic malignancy	27 (23.27)	20 (66.66)	<0.001		3.63 (1.18–11.17)	0.024
Leukemia	12 (10.34)	10 (33.33)	<0.001			
HSCT	6 (5.17)	10 (33.33)	<0.001			
Monocytopenia, <100 cells/μL	26 (22.41)	13 (43.33)	0.021			
Any corticosteroids†	60 (51.72)	25 (83.33)	0.002			
Intensive care unit stay	42 (36.21)	17 (56.66)	0.042			
Mechanical ventilation	17 (14.66)	10 (33.33)	0.019		3.96 (1.16–13.51)	0.028
Presence of a central line	101 (87.06)	30 (100)	0.047			
Azole exposure†	24 (20.68)	20 (66.66)	0.001		5.09 (1.66–15.64)	0.004
Echinocandin exposure†	17 (14.65)	15 (50)	<0.001			
Echinocandin resistance	6 (5.17)	9 (30)	<0.001		5.23 (1.31–20.78)	0.019

*Blank cells indicate that the respective variables did not contribute significantly and were not retained in the final multivariate model (p>0.1). HSCT, hematopoietic stem cell transplantation.  †Within 1 month before the day of candidemia (day of blood collection for culture).

### Echinocandin Resistance

Of the 146 isolates, 24 (16.4%) were intermediate and 15 (10.3%) were resistant to caspofungin. On the basis of the 2008 break point of ≤2 mg/L, 11 (73.3%) of the 15 resistant isolates and 35 (90%) of the 39 intermediate or resistant isolates would have been considered susceptible (*16*). Of 11 caspofungin-resistant isolates that were available for repeat testing, 10 were also resistant to micafungin or anidulafungin. One caspofungin-resistant isolate was intermediate to micafungin and susceptible to anidulafungin (online Technical Appendix Table 1, http://wwwnc.cdc.gov/EID/article/20/11/14-0685-Techapp1.pdf). Factors independently associated with caspofungin resistance were recent echinocandin exposure, total parenteral nutrition (TPN), and monocytopenia (absolute monocyte count <100 cells/μL, Table 3) or severe lymphopenia (absolute lymphocyte count <100 cells/μL [online Technical Appendix Table 2]).

**Table 3:** Factors present at the time of candidemia and associated with caspofungin resistance in cancer patients with *Candida glabrata* fungemia, MD Anderson Cancer Center, Houston, Texas, USA, March 2005–September 2013*

Factor	No. (%)				p value		Multivariate analysis	
	Susceptible (n = 107)	Intermediate (n = 24)	Resistant (n = 15)				Odds ratio (95% CI)	p value
Hematologic malignancy	27 (25.23)	12 (50)	8 (53.33)		.012			
Leukemia	12 (11.21)	4 (16.66)	6 (40)		.012			
Hematopoetic stem cell transplantation	6 (5.61)	5 (20.83)	5 (33.33)		.013			
Neutropenia (<500 cells/μL)	16 (14.95)	6 (25)	6 (40)		.016			
Lymphopenia (<500 cells/μL)	59 (55.14)	15 (62.5)	12 (80)		.069			
Monocytopenia (<100 cells/μL)	20 (18.69)	10 (41.66)	9 (60)		<0.001		3.53 (1.44–8.65)	.006
Mechanical ventilation	17 (15.89)	3 (12.5)	7 (46.66)		.024			
Any corticosteroids†	56 (52.33)	17 (70.83)	12 (80)		.004			
Total parenteral nutrition	22 (20.56)	5 (20.83)	9 (60)		.005		3.37 (1.37–8.24)	.008
Echinocandin exposure†	15 (14.02)	6 (25)	11 (73.33)		<0.001		2.75 (1.09–6.95)	.032
Fluconazole resistance	15 (14.02)	6 (25)	9 (60)		<0.001		3.16 (1.13–7.88)	.013

### Multidrug Resistance

Caspofungin resistance (MIC ≥0.5 mg/mL) was independently associated with fluconazole resistance (Tables 2,3). Among 44 isolates with recent (within 1 month) azole exposure, fluconazole resistance was found for approximately one-third (13 [35.1%] of the 37) that were caspofungin intermediate or susceptible and for all 7 (100%) that were caspofungin resistant (p = 0.002). Among 102 isolates without recent azole exposure, fluconazole resistance was found for 8 (8.5%) of 94 that were caspofungin intermediate or susceptible and for 2 (25%) of 8 that were caspofungin resistant (p = 0.17).

Fluconazole resistance was also independently associated with caspofungin resistance. Among 32 isolates with recent echinocandin exposure, caspofungin resistance was found for 3 (17.6%) of 17 fluconazole dose–dependent isolates and for 8 (53.3%) of 15 fluconazole-resistant isolates (p = 0.034). Among 114 isolates without recent echinocandin exposure, caspofungin resistance was found for 3 (3%) of 99 fluconazole dose-dependent isolates and for 1 (6.7%) of 15 fluconazole-resistant isolates (p = 0.516).

A total of 10 (6.8%) isolates exhibited multidrug resistance (*9*); 2 exhibited in vitro resistance to amphotericin B, 9 exhibited resistance to caspofungin and fluconazole, and 1 was resistant to caspofungin and amphotericin B. Multidrug resistance was found for 30% of fluconazole-resistant strains and 66.6% of caspofungin-resistant strains. All 7 multidrug-resistant isolates that were available for testing were also resistant to micafungin and/or anidulafungin. We did not observe any significant increase in the rates of fluconazole, echinocandin, or multidrug resistance over the 8-year study.

In a separate analysis comparing multidrug-resistant isolates with other isolates, recent (within 1 month before the day of candidemia) echinocandin exposure and TPN were independently associated with multidrug resistance. Values for recent echinocandin exposure were adjusted odds ratio (aOR) 39.9, 95% CI 4.61–345.73, p = 0.001 when compared with all other isolates and aOR 57.22, 95% CI 6.32–517.93, p<0.001 when compared with fluconazole-intermediate and caspofungin-susceptible isolates. Values for TPN were aOR 7.32, 95% CI 1.5–32.83, p = 0.014 when compared with all other isolates and aOR 4.58, 95% CI 0.8–26.26, p = 0.088 when compared with fluconazole-intermediate and caspofungin-susceptible isolates.

In additional analyses, we entered antifungal exposure within 1 year instead of 1 month as an independent variable, and we entered cumulative doses of drug within 1 month or 1 year before the date of candidemia either as continuous or categorical (above vs. below the mean for all patients or those with prior antifungal exposure) independent variables. All associations remained significant, and no increase in predictive value was found for any model.

### Resistance without Prior Exposure to Antifungal Drugs

A total of 11 *C. glabrata* isolates with no documented exposure to the respective classes of antifungal drugs were classified as resistant. A total of 8 isolates with no documented azole exposure were resistant to fluconazole. One of those 8, and 3 additional isolates, were classified as caspofungin resistant, without any documented exposure to echinocandins. Of those 4, the caspofungin MIC was 0.5 mg/L for 3, all of which were fluconazole dose-dependent, and 8 mg/L for 1, which was multidrug resistant. Of those 4 strains, 3 were available for testing of susceptibility to other echinocandins (the multidrug-resistant isolate was not available); 2 were resistant to either micafungin or anidulafungin, and 1 was intermediate to micafungin and anidulafungin. Classification of that 1 isolate as intermediate did not change the results.

### All-Cause Mortality Rates

The 28-day all-cause mortality rate was 39.7% (58/146) among all patients, and 32.3% (30/93) among those who received echinocandins. There was no association between death (log-rank p>0.2) and age (≥65 vs. <65 years), type of malignancy (solid vs. hematologic), or TPN. Among all patients (Technical Appendix Figure) and among the 93 who received echinocandins, caspofungin MIC was inversely associated with 28-day survival rate. Specifically, among patients who received echinocandins, the 28-day crude mortality rates were 25.4% (17/67), 41.7% (5/12), 50% (5/10), and 75% (3/4) for those with isolates with echinocandin MICs of ≤0.125, 0.25, 0.5, and >2 mg/L (the 2008 CLSI break point) (*16*), respectively (log-rank p = 0.001 for linear trend, Figure).

**Figure:**
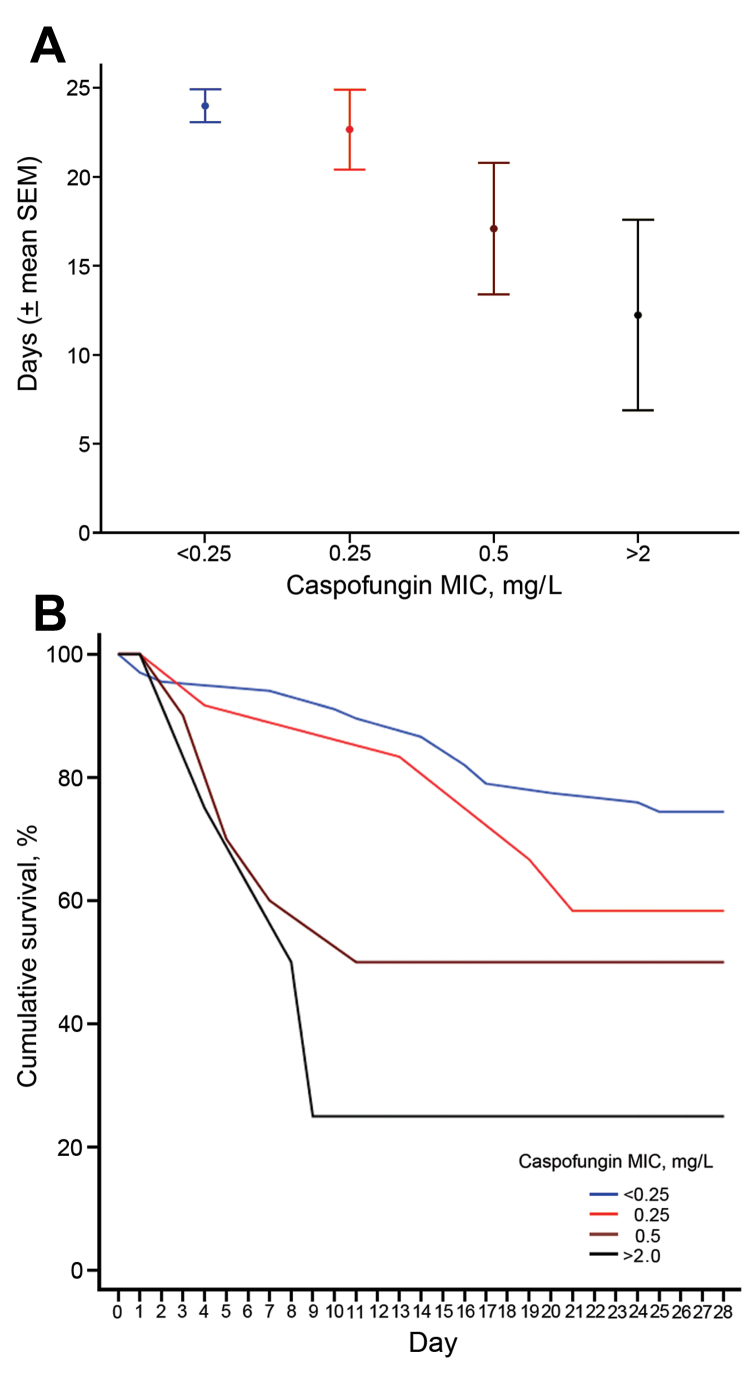
A) Mean 28-day survival (days, mean ± SE) and B) Kaplan-Meier survival curves, relative to caspofungin MIC and susceptibility in *Candida glabrata* isolates, according to the updated definitions (susceptible: MIC<0.25 mg/L, intermediate: MIC = 0.25 mg/L, resistant: MIC ≥0.5 mg/L) and previous definitions (susceptible: MIC ≤2 mg/L, nonsusceptible: MIC >2 mg/L) among 93 patients who received an echinocandin, MD Anderson Cancer Center, Houston, Texas, USA, March 2005–September 2013; log-rank p = 0.001 for linear trend.

Among patients who received echinocandins, the association between caspofungin MIC and all-cause mortality rates remained significant (adjusted hazards ratio [aHR] for MIC ≥0.5 mg/L = 2.59, 95% CI 1.08–6.19, p = 0.033) after adjustment for intensive care unit stay (aHR = 3.8, 95% CI 1.71–8.45, p = 0.001) and monocytopenia (aHR = 4.02, 95% CI 1.89–8.55, p<0.001). Those associations remained significant after reclassification of 1 isolate as intermediate; that isolate was resistant to caspofungin, intermediate to micafungin, and anidulafungin (Technical Appendix Table 1).

## Discussion

In this contemporary series of cancer patients with *C. glabrata* fungemia, the rates of in vitro caspofungin resistance and multidrug resistance are among the highest reported to date. By comparing the updated (*10*) with the previous, non–species-specific CLSI definitions of in vitro susceptibility (*16*), we found that 90% of caspofungin-intermediate or -resistant *C. glabrata* bloodstream isolates would have been previously classified as susceptible. Caspofungin resistance was associated with previous exposure to echinocandins, use of TPN, and all-cause mortality rate.

Contrary to previous findings from our institution (*3*), most patients with *C. glabrata* fungemia in the series reported here had solid tumors rather than hematologic malignancies. One third of fluconazole-resistant isolates and half of those with decreased susceptibility to caspofungin were isolated from patients with solid malignancies. These results probably reflect an overall increase in solid tumors; however, our findings also confirm that *C. glabrata* bloodstream infections have become major clinical problems among all patients at risk for candidemia (*6*,*9*,*14*,*15*,*17*).

In agreement with previously reported findings, our study indicated that broad use of azoles—mainly voriconazole—and echinocandins was strongly associated with *C. glabrata* fluconazole and caspofungin resistance (*3*,*5*,*6*,*14*,*15*). In our study, 11 *C. glabrata* isolates were classified as resistant without having had any previous documented exposure to the respective classes of antifungal drugs. This finding is in agreement with a recent report of isolation of 4 *C. glabrata* FKS mutants from patients who had not received echinocandins (*17*). Because several factors place cancer patients at risk for candidemia and clinical failure of antifungal drugs (*1*–*5*), we sought to identify those clinical factors associated with in vitro resistance. On the basis of our results, we consider it likely that poor host defense mechanisms associated with the presence of hematologic malignancy, myelosuppression, and critical illness are independently associated with resistance. 

We also observed an independent association between TPN and caspofungin resistance or multidrug resistance. TPN is an established risk factor for candidemia and a marker of intestinal dysfunction (*18*). Moreover, TPN causes atrophy of the intestinal mucosa, facilitating microperforations and *Candida* translocation, and it is associated with thick biofilm formation and catheter-related infections (*18*,*19*). Whether our observed association between TPN and caspofungin resistance is reflective of critical illness or whether the above mechanisms also promote the development of resistance remains to be determined.

In our study, almost one third of fluconazole-resistant strains and two-thirds of caspofungin-resistant strains were multidrug resistant. These rates of cross-resistance are significantly higher than those previously reported from multi-institutional registries (*20*,*21*) and another tertiary academic hospital (*6*). Specifically, investigators from Duke University Hospital reported a 25% rate of fluconazole resistance over a 10-year period, which is similar to our rate of 21%. In the same report (*6*), the overall rate of resistance to at least 1 echinocandin was lower (6.7%) than that found in our study (10.7%), although by 2010 it had increased to 12.7% (*6*). In another study, 11% of *C. glabrata* bloodstream isolates were resistant to caspofungin and 18% had FKS mutations (*17*). Notably, the rates of multidrug resistance determined by the study from Duke (3.5%) (*6*), the Centers for Disease Control and Prevention SENTRY Antimicrobial Surveillance Program (1%) (*20*), and another recent multi-institutional study (1%) (*21*) were substantially lower than the rates of multidrug resistance determined in our study (6.8%). These data document a worrisome trend for concomitant resistance of *C. glabrata* clinical isolates to azoles and echinocandins, which seems to be more prominent in our population of patients with cancer.

In our study, resistance to fluconazole was highly associated with caspofungin resistance, independent of prior use of antifungal drugs; this finding is in agreement with our institution’s previously reported findings for different *Candida* species (*22*). Echinocandin, but not azole, exposure was a significant independent predictor of multidrug resistance. These findings could reflect a worrisome potential for development of multidrug resistance in *C. glabrata,* a versatile, haploid species (*7*). In a recent study, serial exposures of a *C. glabrata* laboratory strain to low-dose micafungin led to the development of a single-point mutation conferring multiazole and echinocandin resistance with preserved virulence (*23*). Moreover, in an analysis of molecular events leading to echinocandin resistance of *C. glabrata* isogenic isolates consecutively obtained from a patient receiving chronic TPN, a multidrug-resistant strain emerged after multiple courses of treatment with caspofungin but no previous azole exposure (*8*). Selective pressure from antifungal drugs, along with other factors, such as chemotherapy (*24*) and broad-spectrum antibacterial drugs (*25*), might lead to the expansion of similar phenotypes.

By applying the updated clinical break points to our patient population, we captured a strong and potentially independent correlation of all-cause mortality rates with in vitro caspofungin MICs but not with other factors classically associated with poor outcomes such as advanced age and hematologic malignancy (*2*,*4*,*5*). Although other residual confounders cannot be ruled out, this finding is in agreement with previously reported significant associations between clinical failure of echinocandins and elevated in vitro echinocandin MICs (*6*,*8*,*14*,*17*). In some animal studies, FKS mutations leading to echinocandin resistance were associated with decreased fitness (*8*,*26*). Nevertheless, a recent study that used an immunocompromised murine model of systemic candidiasis showed that caspofungin was ineffective against *C. glabrata* isolates with MIC ≥1 mg/L (*27*). Furthermore, investigators have also described the development of compensatory mechanisms that override the decreased virulence resulting from clinical exposure of an FKS mutant *C. glabrata* isolate to an echinocandin (*8*). Clinical (*8*,*28*) and laboratory (*23*) strains that exhibit high-level antifungal resistance without decreases in fitness have been described. What remains incompletely characterized are the spectrum of mutations predisposing to azole and/or echinocandin resistance, the role of epigenetic mechanisms, and the virulence of resistant (compared with susceptible) *Candida* strains in humans. According to our results, lowering the MIC break point for caspofungin resistance in *C. glabrata* bloodstream isolates to 0.5 mg/L is clinically relevant.

Our study has several limitations. It was a retrospective study performed at a single institution, and our patient population was rather small and selected. Therefore, our observations might not be applicable to different patient groups at risk for serious *Candida* infections. The number of caspofungin-resistant isolates was small, and we used in vitro caspofungin MIC alone to define echinocandin resistance, without molecular confirmation of underlying mutations. The interlaboratory variability in caspofungin MICs is substantial, (*29*,*30*), and there is evidence that micafungin and anidulafungin MICs correlate better with the presence of FKS mutations and clinical outcomes (*15*). However, testing the micafungin and anidulafungin MICs of available caspofungin-resistant isolates did not change our conclusions. Moreover, our most striking finding was the high percentage of multidrug-resistant *C. glabrata* isolates. In a previous study (*20*), 100% of such multidrug-resistant isolates had an FKS mutation; in the study reported here, all multidrug-resistant isolates that were available for testing were resistant to 2 echinocandins. Therefore, we believe that the substantial number of multidrug-resistant strains harbored molecular mechanisms of resistance. It should be noted that the reference for assessing sensitivity and specificity of in vitro MICs has been the presence of mutations within the FKS1 and FKS2 hot spot regions. Nevertheless, there is emerging evidence that non–FKS-related mechanisms might be operative or might predispose to the development of echinocandin resistance and even multidrug resistance (*8*,*23*). Recently, a high in vitro caspofungin MIC (≥0.5 mg/L) was shown (*17*) to have a higher positive predictive value for echinocandin failure than the presence of FKS hot spot mutations, in agreement with our findings and contrary to previously reported findings (*6*,*31*).

In summary, the rate of in vitro caspofungin and multidrug resistance of *C. glabrata* bloodstream isolates in our patient population is, to our knowledge, among the highest reported. Our findings might indicate a worrisome propensity of *C. glabrata* strains for multidrug resistance in cancer patients and should prompt awareness of the need for good stewardship of antifungal drugs. Prospective, large-scale clinical registries, with molecular data on mutations that confer resistance to antifungal drugs, are needed.

Technical Appendix Characteristics of caspofungin-resistant *Candida glabrata* isolates; univariate analysis and multivariate ordinal regression model of factors associated with caspofungin resistance; and Kaplan-Meier survival curves relative to caspofungin MIC and susceptibility in *Candida glabrata* isolates among 146 patients at MD Anderson Cancer Center, Houston, Texas, USA, March 2005–September 2013.
