# Supplementary material for: Drug-Resistant Candida glabrata Infection in Cancer Patients
Source: Emerg Infect Dis. 2014 Nov;20(11):1833–40. doi: 10.3201/eid2011.140685 (PMC4214312; doi:10.3201/eid2011.140685)
Supplement: Technical Appendix — Characteristics of caspofungin-resistant Candida glabrata isolates; univariate analysis and multivariate ordinal regression model of factors associated with caspofungin resistance; and Kaplan-Meier survival curves relative to caspofungin MIC and susceptibility in Candida glabrata isolates among 146 patients at MD Anderson Cancer Center, Houston, Texas, USA, March 2005–September 2013. [file 14-0685-Techapp-s1.pdf]

Article DOI: http://dx.doi.org/10.3201/eid2011.140685

Drug-Resistant *Candida glabrata* Infection in Cancer Patients

Technical Appendix

Technical Appendix Table 1. Characteristics of caspofungin-resistant *Candida glabrata* isolates*

Isolate no.	MIC (mg/L)			MDR	Documented echinocandin preexposure†
	Caspofungin	Micafungin	Anidulafungin		
5‡	0.5	0.125	0.25	-	-
12	0.5	0.125	0.5	-	-
15	0.5	0.25	0.25	+	+
21	4	NA	NA	+	+
51	0.5	0.25	0.5	-	-
59	4	0.5	0.5	+	+
61	0.5	0.25	0.25	+	+
66	8	0.5	1	+	+
106	0.5	0.25	0.25	+	+
107	8	NA	NA	+	-
112	0.5	0.25	0.25	+	+
122	0.5	0.25	0.25	-	+
126	0.5	NA	NA	-	+
131	0.5	NA	NA	+	+
136	0.5	0.06	1	+	+

*MDR, multidrug resistant; NA, not available for testing.
†Within 1 year before the day of candidemia.
‡All parameters associated with antifungal resistance remained significant if that isolate was reclassified as intermediate. Among patients treated with echinocandins, the association between caspofungin MIC and all-cause mortality rate also remained significant (adjusted hazard ratio [aHR] for MIC ≥0.5 mg/L: 3.17 [95% CI 1.26-8.01], p =.015), after adjustment for ICU stay (aHR: 4.03 [95% CI 1.79-9.07], p =.001) and monocytopenia (aHR: 3.67 [95% CI 1.69-8], p =.001).

Technical Appendix Table 2. Univariate analysis and multivariate ordinal regression model of factors associated with caspofungin resistance, using severe lymphopenia instead of monocytopenia*

Factor	No. (%)				Multivariate analysis		
	Susceptible (n = 107)	Intermediate (n = 24)	Resistant (n = 15)	p value	Odds ratio	95% CI	p value
Severe lymphopenia (<100 cells/μL)	15 (14)	6 (25)	9 (60)	<.001	3.23	1.29-8.03	.012
TPN	22 (20.56)	5 (20.83)	9 (60)	.005	3.01	1.28-7.11	.012
Echinocandin exposure†	15 (14.02)	6 (25)	11 (73.33)	<.001	2.97	1.19-7.38	.019
Fluconazole resistance	15 (14.02)	6 (25)	9 (60)	<.001	3.17	1.27-7.88	.013

*TPN, total parenteral nutrition.
†Within 1 month before the day of candidemia.

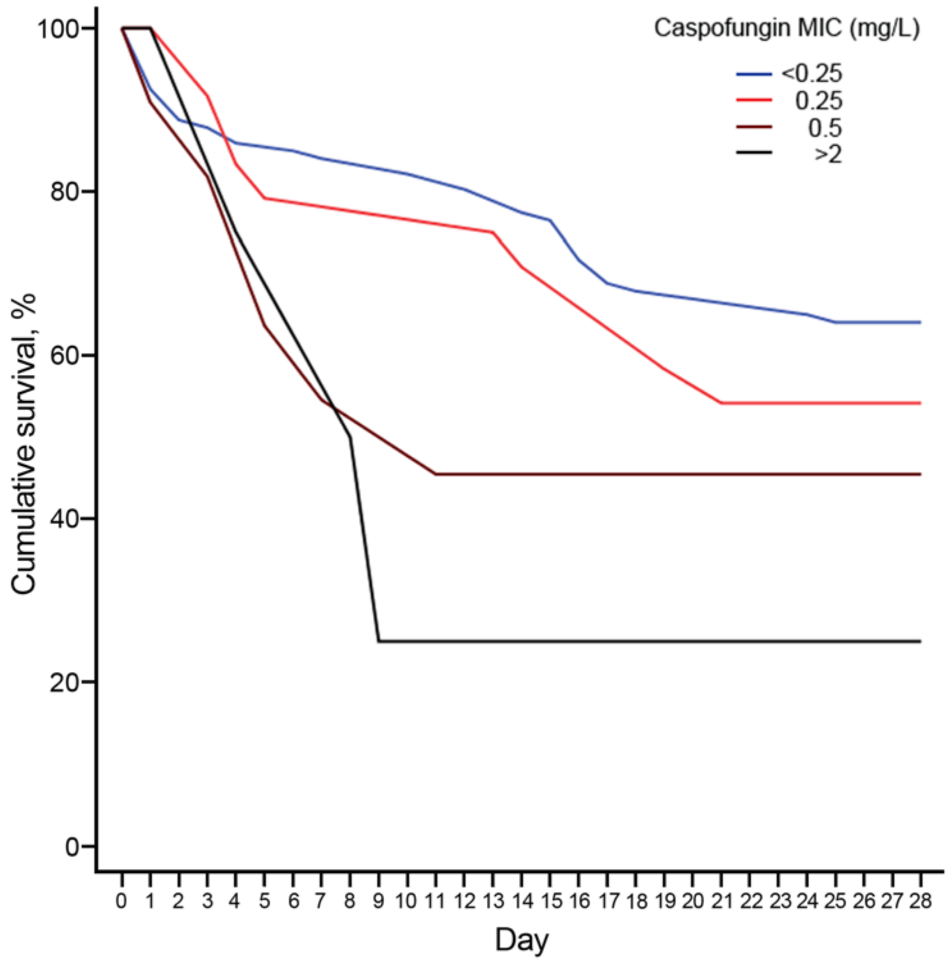

Technical Appendix Figure. Kaplan-Meier survival curves, relative to caspofungin MIC and susceptibility in *Candida glabrata* isolates, according to the updated definition (susceptible: MIC<0.25 mg/L, intermediate: MIC = 0.25 mg/L, resistant: MIC ≥0.5 mg/L) and previous definitions (susceptible: MIC ≤2 mg/L, nonsusceptible: MIC >2 mg/L), among all 146 patients; log-rank p = 0.002 for linear trend.
